# Linking the Divergent and Convergent Processes of Collaborative Creativity: The Impact of Expertise Levels and Elaboration Processes

**DOI:** 10.3389/fpsyg.2019.00699

**Published:** 2019-04-12

**Authors:** Lauren E. Coursey, Ryan T. Gertner, Belinda C. Williams, Jared B. Kenworthy, Paul B. Paulus, Simona Doboli

**Affiliations:** ^1^Department of Sociology and Psychology, University of North Texas at Dallas, Dallas, TX, United States; ^2^Hollweg Assessment Partners, Dallas, TX, United States; ^3^Department of Psychology, University of Texas at Arlington, Arlington, TX, United States; ^4^Department of Computer Science, Hofstra University, Hempstead, NY, United States

**Keywords:** expertise, innovation, diversity, elaboration, divergent creativity, convergent creativity, fixation

## Abstract

We examined the impact of task-relevant expertise level in groups on the idea sharing and elaboration process and on idea development. Participants were assigned to low, heterogeneous, and high expertise groups and were asked to generate ideas for the development of a new sport. Following two asynchronous divergent ideation phases using an electronic discussion board for ideational exchanges, groups completed a synchronous convergent discussion phase in which they selected and refined their ideas for a new sport. The number of ideas and their novelty during the divergent phase did not influence the outcome of the convergent phase. However, consistent with our theoretical model final product novelty was influenced by the number and novelty of the replies in the divergent phase. Although group expertise level was associated with various performance outcomes in the divergent ideation phase, it did not impact the novelty of the final product. Low expertise groups demonstrated the most novelty in the divergent phase. Final product novelty was also associated with sports words used in discussions during the convergent phase.

## Introduction

Collaborative ideation or creativity is an important factor in the innovative progress of organizations and countries, and for the past 30 years much research has focused on this process (Paulus and Nijstad, 2003, 2019; Puccio, 2017; Graesser et al., 2018; Paulus et al., 2018; Reiter-Palmon, 2018). Much of the past research has focused almost exclusively on the divergent processes that contribute to idea generation (Cropley, 2006; McMahon et al., 2016). Divergent ideation is integral to creativity, but real-world innovation requires the type of convergent synthesis rarely studied in laboratory groups (Harvey, 2014). There is also very little research concerning the links among the various phases of the collaborative innovation process—ideation, elaboration, evaluation, selection, and development or implementation (e.g., de Vries and Lubart, 2017; Puccio et al., 2018; Rosing et al., 2018).

The goal of the present study was to examine the role of the group interaction and ideation processes within an experimental design that varied the expertise level of groups that interacted across both the divergent and convergent creativity stages of a “naturalistic” idea exchange paradigm. We used an electronic discussion board to allow groups to interact asynchronously for 2 weeks by generating ideas, voting on the ideas, replying, and elaborating – what we will term *divergent* creativity. This was followed by a synchronous, *convergent* discussion session in which groups were asked to interact in real time to evaluate previous ideas and come up with a final product based on their prior exchanges. Previous research has not examined the link between the group discussion interaction processes and outcomes in the divergent phase to the outcomes of the convergent, innovation phase. We will present the empirical and theoretical background for this study from the perspective of the group creativity literature.

### The Divergent to Convergent Innovation Model (DCIM)

**FIGURE 1:**
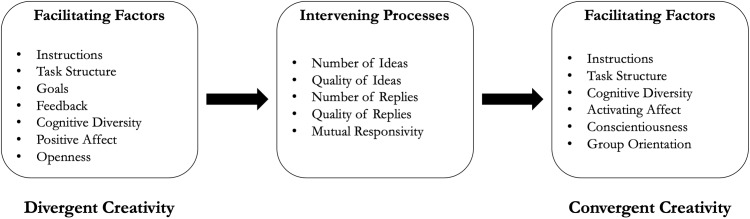
A theoretical model of factors facilitating divergent and convergent collaborative creativity, including intervening processes linking divergent and divergent creativity.

To frame the research agenda for a study of the links between the divergent and convergent creativity processes we have developed a model in which we highlight the key factors that should influence the divergent and convergent processes, and those factors that should influence the link between them (Paulus et al., 2019). A version of the DCIM is shown in Figure 1. Research has found that the divergent process of generating ideas in groups is influenced by the use of appropriate instructions, task structure (category focus, breaks), goals, feedback, cognitive diversity, positive affect, and openness to experience (cf., Paulus et al., 2018; Nijstad et al., 2019; Paulus and Kenworthy, 2019). Although there is not much research on convergent collaborative creativity to guide our list of facilitating factors (cf., Rietzschel et al., 2010, 2019), the literature has provided some basis for predictions. Appropriate instructions and task structure (e.g., Lonergan et al., 2004; Putman and Paulus, 2009), cognitive diversity (Larey and Paulus, 1999; Cronin and Weingart, 2007), some degree of negative affect related to increased task persistence (George and Zhou, 2002; Zhou and Su, 2010; To et al., 2012), conscientiousness (Fürst et al., 2016) and a positive orientation to groups (Larey and Paulus, 1999) all may be related to more effective evaluation, selection, and building processes.

A key focus of this study is the processes that connect the divergent and convergent phases. That is, what divergent processes influence the quality of the final product developed in the convergent phase? Obviously, the number and quality of ideas generated should have some impact on the final product since they provide the basic foundation for the evaluation and selection processes in the convergent phase. However, our prior research and that of others has highlighted the importance of the elaboration processes (Mesmer-Magnus and DeChurch, 2009; van Knippenberg, 2017; Coursey et al., 2018). That is, to what extent do group members elaborate on the shared ideas and make them more novel? In the present study we focused on the role of these process in addition to examining the role of cognitive diversity.

The elaboration process has been emphasized as important for the effective realization of creative potential in diverse groups (van Knippenberg et al., 2004; Harvey, 2015). Elaboration can be defined as “the exchange of information and perspectives, individual-level processing of the information and perspectives, the process of feeding back the results of this individual-level processing into the group, and discussion and integration of its implications” (van Knippenberg et al., 2004, p. 1011). Research by van Knippenberg and colleagues has highlighted the importance of information elaboration in groups for the enhancement of innovation as well as for optimal group decision-making (e.g., van Dijk and van Knippenberg, 2008). Kohn et al. (2011) found that group exchange processes may be particularly useful for building on relatively rare ideas to come up with additional novel and useful ideas.

A few studies have examined the idea selection process after idea generation. Generally speaking, groups are not particularly good at picking the best ideas from the prior divergent thinking session, and there is a tendency for groups to choose ideas that are not novel (Rietzschel et al., 2006; Putman and Paulus, 2009; Mueller et al., 2011). This suggests that there may not be a strong link between the quality of the idea generation phase and the development of a specific innovation (Harvey and Kou, 2013).

Thus, one goal of this study was to examine the link between the divergent and convergent processes of creativity. The ability of a group to identify the best ideas and to build on them within the convergent phase is essential for a final creative product. This is a complex collaborative task that can be associated with a high level of cognitive load as group members deal with a large pool of ideas from the divergent stage. In this study, we examined the impact of both group expertise level and the interaction processes during the divergent stage on the quality of the group’s discussion and final product which took place following the divergent phase.

We employed a methodology that allowed participants to build on each other’s ideas by voting, quoting, replying, and integrating, so that we could examine the relationship of elaboration processes to outcomes in both the divergent, ideation phase and the convergent, discussion and innovation phase.

#### Group Diversity and Group Creativity

There is a growing body of research addressing the impact of diversity on group, or team, creativity (Bell et al., 2011; Paulus and Coskun, 2012). It is now clear that group interaction dynamics can be an important basis for the generation of creative ideas. Furthermore, collaborative creativity can be facilitated by a diverse group composition (van Dijk et al., 2012; Paulus and van der Zee, 2015). Demographic differences such as race, ethnicity, and gender can enhance the performance of groups if the diverse characteristics are related to task-relevant dimensions (Bantel and Jackson, 1989; van Knippenberg and Schippers, 2007; Woolley et al., 2010; Nakui et al., 2011). For example, Bantel and Jackson (1989) found that, whereas age and tenure were negative predictors of banking innovation, both higher education and the functional diversity of banking teams were positive predictors of innovation. In Woolley et al. (2010), the proportion of females in groups was a strong predictor of those groups’ collective intelligence and performance, primarily because gender was associated with increased social sensitivity.

Past research has focused on both demographic diversity, as well as expertise, or functional, diversity. In the case of expertise diversity, the presumed diversity in relevant knowledge, perspectives, and experience should provide a basis for enhanced collaborative creativity and innovation. However, to date, researchers have not investigated the effects of systematic variations in levels of expertise on the processes leading to collaborative creativity.

#### Expertise Heterogeneity

Although many work teams or groups may consist of people with unique areas of expertise, on any particular task they will also vary in level of expertise. In many contexts, the variation in level of expertise may be the primary factor differentiating potential contributions to the group or team task. For example, research team members focusing on a particular issue may have a similar educational background, but differ in their level of expertise or experience in their area of study. The impact of level of expertise on collaborative creativity has not been examined independent of variations in areas or types of expertise and will be a focus of this study.

There is considerable evidence in the creativity literature that expertise in a particular domain is related to creativity in that specific domain (Ericsson and Ward, 2007; Boh et al., 2014; Ericsson, 2014; Baer, 2015; Simonton, 2015). Having some knowledge and experience in a domain will provide the knowledge base from which creative ideas can be developed (Nijstad and Stroebe, 2006). Furthermore, expertise has been shown to enhance divergent thinking, as in the case of expertise in military leaders (Vincent et al., 2002). Yet, having high levels of expertise can also increase the degree of inflexibility in thinking (Sternberg, 1996; Lewandowsky et al., 2007), potentially related to the stability of their cognitive domain schemas (Dane, 2010). This relationship has also been demonstrated in the problem-solving literature (e.g., Jansson and Smith, 1991; Bilalic et al., 2008). Wiley (1998) found that domain expertise inhibited individual performance on a creative problem-solving task. Dane (2010) has suggested that the domain knowledge of experts may increase their ability to develop ideas that are incremental in the level of novelty, but may limit their ability to generate more radically creative ideas.

To date, there is no known research that has provided a clear examination of the expertise issue in the context of collaborative creativity, particularly in cases where groups differ systematically in their degree of expertise on a single dimension (such as expertise in physics, sports, music, architecture, etc.). In this type of task context, one can clearly examine the differential effects of degree of expertise independent from type of expertise. In the present study, we compared the creativity of groups that varied experimentally in their respective levels of expertise—low, high or mixed—to determine the degree to which group-level expertise facilitates or hinders creativity.

### Overview and Hypotheses

Based on pilot work, the task for this study was to come up with ideas for the creation of a new sport. Our experimental design consisted of three experimental conditions that varied in their average level of expertise (low, heterogeneous, and high) with respect to the topic of the idea generation task. The groups first generated ideas electronically in an asynchronous fashion, then the groups came together in a synchronous, discussion session by means of an audio Skype call, allowing members to discuss the creation of a new sport, based on their previously shared ideas.

On the basis of the DCIM model (see Figure 1) and research highlighting the importance of elaboration of shared ideas (van Knippenberg et al., 2004; Kohn et al., 2011; Coursey et al., 2018), it is expected that the degree of elaboration of ideas (replies, etc.) will be associated with increased novelty of the final products. Elaboration suggests a high degree of cognitive involvement which should increase the quality of the shared ideas (Nijstad and De Dreu, 2012) and facilitate a later consensus on the ideas to incorporate into a final product.

*Hypothesis 1.* Across group types, the number of replies during the idea generation stage will be related to increased novelty of the final products.

We also expect that the novelty of ideas in the divergent ideation phase will be associated with the novelty of the final product. It is presumed that the groups that generate more novel ideas will be able to come up with a more novel sport in the convergent, group discussion phase. However, because groups and individuals have a tendency not to select the most novel ideas as their best ideas (Rietzschel et al., 2006; Putman and Paulus, 2009; Mueller et al., 2011), it is possible that there may not be such a straightforward connection between the divergent phase novelty and the development of a convergent, final product. It is likely that only novel ideas that have involved active collaboration will be incorporated into a final product. A key indicator of collaborative involvement is the extent to which ideas elicit novel replies (Coursey et al., 2018). The novelty of the replies reflects the degree of task motivation and engagement, attention to others’ ideas, and the extent of cognitive stimulation, enhancement, and integration of the shared ideas. This should heighten the salience of certain ideas for possible inclusion in a final product. Thus, reply novelty is likely to be associated with greater novelty of the final product.

*Hypothesis 2.* Across group types, the novelty of replies during the divergent phase will be associated with final product novelty.

In the final convergent, product development phase, group members propose which of the shared ideas should be included in the final product. The content of the discussion may be critical to the final outcome. High expertise individuals are likely to focus on more conventional expertise-related content, but low expertise individuals are more likely to include non-conventional elements in their discussion. The use of conventional product-related words may limit the novelty of the final product developed by the high expertise groups.

Although one might expect high levels of expertise to be related to creativity (Vincent et al., 2002), as noted above, expertise can also limit one’s ability to generate novel perspectives due to cognitive fixation (Wiley, 1998). Those with a high degree of expertise can share their knowledge about a domain with the group, but their expertise may hinder the development of truly novel ideas. They may self-censor novel ideas because of low perceived feasibility. Low expertise individuals, by contrast, will have little cognitive constraint on their ideas related to a particular domain. On the broad-based and open-ended divergent thinking task employed in this study, high levels of expertise may inhibit group creativity. That is, the specific case-based knowledge may inhibit the ability of high-expertise groups to generate novel ideas.

*Hypothesis 3.* High expertise groups will generate fewer ideas, fewer replies, and fewer novel ideas, compared to low expertise groups and heterogeneous groups.

*Hypothesis 4.* High expertise groups are expected to use more conventional product-related words during final product development phase and the use of such words should moderate the relationship between expertise and final product novelty. We predict that a focus on conventional sports will be detrimental to high expertise groups. Specifically, we expect that when conventional word usage is high, high expertise groups will have lower final product novelty compared to low expertise groups.

Although the level of expertise can influence creativity, the degree of diversity in expertise may also be important. A group that is diverse in level of expertise may generate the most novel ideas because the low expertise individuals are not constrained by domain-specific expertise. High expertise members can use their knowledge and experience to build on low expertise group members’ ideas (Dunbar, 1997), perhaps rendering truly outlandish ideas more feasible. Past research indicates that diversity in expertise can lead group members to more deeply process information leading to unique idea generation (Harvey, 2015), increased range of information availability (van Knippenberg and Schippers, 2007), enhanced divergent creativity (Rietzschel et al., 2007), and even enhanced team performance (Paulus and van der Zee, 2015). Alternatively, differences in expertise and interest level that are too great may inhibit the idea sharing and elaboration processes. Cronin and Weingart (2007) suggest that cognitive and representational gaps in diverse groups can have negative effects on group outcomes. Due to the mixed findings in the literature, we offer the following competing hypotheses.

*Hypothesis 5a.* The heterogeneous expertise groups will generate more ideas, more replies, and more novel ideas compared to the other group types.

*Hypothesis 5b.* The heterogeneous group will perform poorly compared to the other groups, because of the cognitive and interest gaps that exist in such groups.

## Materials and Methods

### Participants and Design

Two hundred thirty participants volunteered to complete an online survey about expertise and interests in sports, as well as to subsequently volunteer in an idea generation task which focused on developing a new sport. Data was lost for one group (due to a clerical error during the sign-in process that corrupted the data), leaving a total sample size of 226 participants. The participants were undergraduate students at a large, public university in the southern United States who were recruited via the psychology department’s participant pool. They participated in exchange for partial course credit. The 7-item prescreening questionnaire (α = 0.86) asked participants to indicate the degree to which they were interested in sports and had past experience playing sports (e.g., “Please rate your interest in sports in general,” “How confident would you be in explaining the rules of your favorite sport to someone who has never seen it before?”, etc.). A 5-point scale was used to rate these statements (1 = *very low*, 5 = *very high*). Mean scores were computed for each participant in order to assess individual level of expertise. Participants were not randomly assigned to expertise levels; rather, based on their self-reported expertise, they were randomly assigned to group composition types (i.e., homogeneous and heterogeneous). Participants scoring above the sample mean on the expertise composite were randomly assigned to either the high expertise composition condition (*k* = 17) or the heterogeneous condition (*k* = 22). Participants scoring below the sample mean on the expertise composite were randomly assigned to either the low expertise composition condition (*k* = 18) or the heterogeneous condition. Our heterogeneous condition included those scoring above and below the mean in various proportions (e.g., a heterogeneous group may contain one person scoring above the mean and three scoring below the mean on expertise, or alternatively it may contain two members scoring above the mean and two members scoring below the mean on expertise). The sample included 153 (67.7%) females and 73 (32.3%) males. The demographic composition included 34.1% White/Anglo-American, 32% Hispanic, 23.9% Asian, 20.8% Black/African American, and 13% other (1.3% did not report). Participants’ ages ranged from 18 to 46 with a mean age of 20.8 years.

### Procedures

Participants were run in groups of four (except for two groups of three, due to attrition). Written informed consent was obtained from each of the participants. Each group met for three sessions over the course of 3 weeks. Both sessions one and two were asynchronous, and group members generated ideas in the laboratory individually, according to their own schedule. Participants were never told to which experimental condition they were assigned. Sessions one and two were considered divergent phases because participants were asked to generate ideas, elaborate, and vote on ideas. Participants were instructed to generate as many ideas and elaborations as possible and were instructed to vote on as many ideas as they wished. On the other hand, session three was considered a convergent phase as participants were instructed to select, and develop further if needed, a single idea that would serve as their group’s “final product.”

#### Session One

In session one, participants generated ideas individually for 30 min on their own separate discussion board, created using Simple Machines Forum software^1^. Participants were given an anonymous username (e.g., 79ab35 or 33ec80) and specific instructions concerning the functions of the message board. The participants were instructed to log in to an online discussion board and generate as many ideas as possible on developing a new sport. In addition, participants were able to quote and build on their own ideas in session one using the discussion board’s quote and reply function. They were given five brainstorming rules (“Criticism is ruled out”; “Freewheeling is welcome”; “Quantity is wanted”; “Stay focused on the task”; “Build on ideas”; Osborn, 1957; Putman and Paulus, 2009). They were also given an operational definition for a sport, namely, an activity involving physical exertion and skill in which an individual or team competes against another or others.

#### Session Two

For this session, all group members’ posts from the first session were combined into a single, group message board. As each group member was assigned an anonymous username (e.g., 79ab35) for each of their posts to the discussion board, cues to their respective identities were avoided. Participants were given 15 min to read and familiarize themselves with the other group members’ ideas and vote on the ideas they believed to be the best or most creative using the phrase “%good idea%.” After voting, participants were asked to elaborate on the existing ideas, as well as to generate new ideas for 30 min. During this phase participants were only able to see group members’ ideas from session one. This procedure is similar to the one used by Kohn et al. (2011) in which participants built on ideas generated in a prior session. Each participant had their own individual discussion board in which they completed idea generation and voting asynchronously.

#### Session Three

In this session (convergent phase), participants from each group returned to the lab during the same time slot, were placed in separate rooms, and the ideas that were posted from sessions one and two were once again combined into a single message board at which point participants could view other members’ posts in real time (synchronously). The participants were instructed to read over the elaborated ideas from session two for 15 min and vote on the ideas they believed to be the best, or most creative, using the same phrase from session two “%good idea%.” Following this, participants were connected via Audio Skype. They were then instructed to create and finalize a new sport, considering the ideas that their group had previously posted from sessions one and two. The method of selecting a final idea was not imposed by the researchers; they were free to come up with their plan in any fashion. Guidelines were provided for the necessary components of the final product. They were told the sport must involve physical movement, physical activity, or an athletic component (see Appendix A for details). Additionally, their ideas should address questions such as “How will your sport be structured?”, “How many players?”, “What object/tools are used?”, “What are the rules going to be?”, and “What are the time duration, physical space dimensions, and determinants of scoring?” Each group was given 30 min to complete their sport and to fill out a separate text document detailing its components.

### Measures

#### Expertise

As described above, a mean score was computed for each individual based on the 7-item prescreening questionnaire for sports expertise. The mean expertise for each condition was as follows: low expertise (*M* = 1.91, *SD* = 0.29), heterogeneous expertise (*M* = 2.51, *SD* = 0.49), and high expertise (*M* = 3.39, *SD* = 0.32). An analysis of variance revealed an omnibus test of group differences, *F*(2,56) = 63.48, *p* < 0.001, and *post hoc* comparisons showed that each of the groups differed from both other conditions at *p* < 0.001.

#### Number of Ideas and Replies

Two independent judges counted the number of original ideas and replies of each participant in the divergent phases. Absolute agreement between judges was required. Total number of ideas and of replies were then calculated at the group level.

#### Number of Votes

The number of votes for each idea and reply throughout the divergent phase required absolute agreement between two independent judges. The number of votes were counted (a) for each individual idea, as well as (b) how many times each group member received votes from other group members, and (c) how many votes each group member cast.

#### Novelty

Each individual idea and reply was coded for novelty on a 5-point scale from 1 (*a very common idea*) to 5 (*a very original/unique idea*) by two independent raters. The two independent raters were blind to condition and unaware of the number of votes each idea received. Novelty is considered solely within the context of the corpus of documents of the experiment. A total, or sum, novelty score was then calculated at the group level with a low score indicating low novelty. Two independent coders rated 25% of the overall ideas. The inter-rater reliability was good (α = 0.86), and one coder rated all remaining ideas. Average novelty scores were created by dividing the groups’ total idea novelty score by the number of ideas for the group and the groups’ total reply novelty score by the total number of replies for the group. Finally, a group-level variable was created to reflect the number (i.e., count) of highly novel ideas and replies. An idea rated as a 4 or 5 by our coders was considered to be highly novel.

#### Linguistic Inquiry Word Count (LIWC)

The Skype conversations were first transcribed into text, then analyzed using the LIWC computerized text software (Pennebaker et al., 2007) to examine the words used in the group discussions. The LIWC program analyzes each separate document in comparison to a pre-existing dictionary file. We analyzed each transcript for the proportion of words fitting within a custom-made dictionary category containing conventional words related to sports (e.g., football, basketball, hit, throw, run, etc.).

#### Final Product Novelty

The final product of the convergent phase was also coded for novelty based on the same 5-point scale discussed for novelty ratings of individual ideas. Two independent raters, blind to experimental condition, rated the final products on the dimension of novelty (ICC = 0.61). The two ratings per group were averaged to create the group’s final product novelty score. Samples of individual ideas, as well as highly novel and highly non-novel final products are provided in Appendix B.

## Results

Descriptive statistics and correlations of study variables are displayed in Table 1. The most consistent predictors of various outcomes were novelty of ideas and of replies. Only novelty of replies had a significant positive relationship with novelty of the final product. Consistent with Hypothesis 4, the number of conventional sports words used during the audio Skype sessions was positively related to mean levels of expertise but negatively related to the novelty of the final product. Due to the correlations between DVs, we formally test hypotheses, below, using multivariate analyses. Because the proportion of females in groups was associated with group-level expertise variability, we modeled it as a covariate in our analyses below. See Table 1 for all intercorrelations.

**Table 1:** Descriptive statistics and intercorrelations among study variables.

Variables	*M*	*SD*	1	2	3	4	5	6	7	8	9
(1) Proportion female	0.68	0.23	–								
(2) Number of ideas	27.23	12.02	-0.26ˆ	–							
(3) Number of replies	19.28	9.00	-0.25ˆ	0.48**	–						
(4) Novelty of ideas	90.95	43.07	-0.25ˆ	0.89**	0.42**	–					
(5) Novelty of replies	48.93	22.67	-0.19	0.43**	0.93**	0.45**	–				
(6) Average novelty of ideas	3.36	0.66	0.03	-0.07	-0.05	0.35**	0.07	–			
(7) Average novelty of replies	2.59	0.46	0.21	-0.21	-0.24ˆ	-0.04	0.09	0.43**	–		
(8) Novelty of final product	2.78	0.82	0.01	0.11	0.29*	0.11	0.31*	-0.09	0.01	–	
(9) Sports content	0.56	0.79	0.00	-0.11	-0.18	0.04	-0.10	0.31*	0.19	-0.35*	–
(10) Group mean age	20.69	1.52	0.13	-0.17	0.10	0.15	0.00	0.13	-0.07	0.02	-0.01


**ˆp < 0.10, ^∗^p < 0.05, ^∗∗^p < 0.001*.*

### Divergent Phase

A MANCOVA was conducted to examine the effects of experimental group composition on group-level performance in the divergent phase. Specifically, this MANCOVA examined the effect of group composition on overall productivity or fluency. The number of ideas and number of replies were entered as multiple dependent measures. Proportion of females in the group was entered as a covariate. Multivariate assumptions of homogeneity of covariance matrices and equality of error variances for step-down analyses were met. The proportion of females in the group significantly predicted the multivariate composite, Wilks’ λ = 0.84, *F*(2,52) = 5.15, *p* = 0.009, $\eta_{p}^{2}$ = 0.17. The multivariate effect of group composition was significant, Wilks’ λ = 0.79, *F*(4,104) = 3.33, *p* = 0.013, $\eta_{p}^{2}$ = 0.11. Roy-Bargmann step-down analyses were performed to test the effect of group composition on individual dependent measures. Group composition significantly predicted number of ideas, controlling for proportion of females, *F*(2,53) = 6.20, *p* = 0.004, $\eta_{p}^{2}$ = 0.19. However, group composition did not significantly predict the number of replies controlling for proportion of females and number of ideas. Pairwise comparisons, using Bonferroni corrections, were performed to further probe significant group composition effects on number of ideas. Low expertise groups generated a significantly greater number of ideas (*M* = 33.52, *SE* = 2.60) than did the heterogeneous groups (*M* = 21.32, *SE* = 2.30), *p* = 0.003; neither of these groups differed significantly from the high expertise groups (*M* = 28.22, *SE* = 2.62).

A second MANCOVA examined the effect of group composition on average idea and reply novelty and total number of highly novel (i.e., those rated as 4 and above) ideas and replies. Multivariate assumptions of homogeneity of covariance matrices and equality of error variances for step-down analyses were met. The proportion of females in the group was again included as a covariate in all step-down analyses. The multivariate effect of group composition was significant, Wilkes’ λ = 0.69, *F*(8,100) = 2.53, *p* = 0.015, $\eta_{p}^{2}$ = 0.17. Roy-Bargmann step-down analyses were performed to test the effect of group composition on individual DVs. In step one, group composition did not significantly predict average idea novelty. In step two, group composition significantly predicted average reply novelty, controlling for average idea novelty, *F*(2,52) = 3.96, *p* = 0.025, $\eta_{p}^{2}$ = 0.13. In step three, group composition significantly predicted number of highly novel ideas, controlling for average idea and reply novelty, *F*(2,51) = 4.68, *p* = 0.014, $\eta_{p}^{2}$ = 0.16. Group composition did not significantly predict number of highly novel replies, controlling for all other DVs. Pairwise comparisons using Bonferroni corrections were performed to further probe significant group composition effects on average reply novelty and number of highly novel ideas. The heterogeneous expertise group had significantly higher average reply novelty (*M* = 2.75, *SE* = 0.09) than the low expertise group (*M* = 2.37, *SE* = 0.10); neither of these differed significantly from the high expertise groups (*M* = 2.66, *SE* = 0.11). Finally, low expertise groups generated a significantly greater number of highly novel ideas (*M* = 16.81, *SE* = 1.52) than did the heterogeneous groups (*M* = 10.50, *SE* = 1.31), *p* = 0.012; neither of these groups differed from the high expertise groups (*M* = 15.16, *SE* = 1.90).

#### Endorsement of Novel Versus Non-novel Ideas

The novelty of ideas and replies was dichotomized as either novel or non-novel. As noted above, ideas that received a novelty rating of 4 or 5 were deemed highly novel whereas ideas with a novelty rating below 4 were non-novel ideas. A chi-square test of independence indicated a significant association between high versus low novelty of ideas/replies and votes versus non-votes for ideas/replies within the divergent phases, across all expertise conditions (low, heterogeneous, and high), *X*^2^(1) = 41.29, *p* < 0.001, Cohen’s *w* = 0.13, Odds Ratio = 1.77 (95% C.I.: 1.49/2.11). This indicates that the proportion of votes to non-votes was higher for novel than for non-novel ideas. Table 2 presents the proportions of voting versus non-voting for novel and non-novel ideas within each of the experimental conditions. As indicated, the effect size (Cohen’s *w*) for this ratio in the high expertise condition is roughly half the magnitude as those in the other two conditions. Examined another way, of the ideas that were rated as highly novel, 38 and 40% of those were voted on in the low expertise and heterogeneous conditions, respectively. By contrast, only 31% of highly novel ideas were voted on in the high expertise condition. This suggests that the tendency to vote for novel versus non-novel ideas was weaker in the high expertise conditions, consistent with our expectations (and Hypothesis 4) that homogeneous high expertise groups would display a comparative affinity for more conventional ideas.

**Table 2:** Crosstabulation of ideas and votes.

Vote category per condition	Votes per type of idea						
	
	Non-novel	Novel	χ2	Cohen’s *w*	Odds ratio	95% Lower C.I.	95% Upper C.I.
**Low expertise**							
No votes	444 (1.4)	182 (-1.9)	19.44**	0.15	1.97	1.45	2.66
Votes	139 (-2.2)	112 (3.0)					
**Heterogeneous**							
No votes	401 (1.6)	187 (-2.1)	21.49**	0.16	2.01	1.49	2.69
Votes	138 (-2.3)	129 (3.1)					
**High expertise**							
No votes	342 (0.7)	222 (-0.8)	4.45*	0.08	1.40	1.02	1.92
Votes	113 (-1.2)	103 (1.4)					


**^∗^p < 0.05, ^∗∗^p < 0.001. Adjusted standardized residuals appear in parentheses, and indicate the measure of strength of the difference between observed and expected values; negative scores indicate that the observed frequency is less than the expected frequency. Odds ratio indicates odds of voting for a novel idea, compared to voting for a non-novel idea*.*

### Divergent to Convergent Phase

Two separate multiple regression analyses were conducted to examine the effects of divergent phase productivity and content on convergent phase final product novelty. These were conducted separately because of the high intercorrelations between the number of ideas and the novelty of ideas, and between the number of replies and the novelty of replies, respectively (see Table 1). The first model examined the effects of the number of ideas and replies on final product novelty, whereas the second model examined the effects of idea and reply novelty on final product novelty. The first full model was not significant, *F*(3,49) = 1.62, *p* = 0.196, adjusted *R*^2^ = 0.04. However, divergent phase number of replies predicted convergent phase final product novelty, β = 0.32, *t*(49) = 2.05, *p* = 0.046; greater number of discussion replies was a significant predictor of final group product novelty. The second full model was not significant, *F*(3,49) = 1.18, *p* = 0.157, adjusted *R*^2^ = 0.05. However, divergent phase reply novelty predicted convergent final product novelty, β = 0.33, *t*(49) = 2.18, *p* = 0.034; greater novelty of discussion replies was a significant predictor of final group product novelty. Taken together, these findings indicate that when group members replied and elaborated on each other’s ideas with a high frequency and in novel ways during the divergent phase, their final products became more highly novel.

### Convergent Phase

We anticipated that there would be differences between the expertise conditions with respect to their final products as a function of their topical content. Specifically, we expected that final product novelty would be lower to the degree that high expertise groups fixated on conventional sports content, but that final product novelty would not vary as a function of topical content for heterogeneous or low expertise groups. Thus, an ANCOVA was conducted in which group type served as the independent variable and proportion of females was included as a covariate to predict final product novelty. There was no main effect of group composition for the rated novelty of the final product (low expertise *M* = 3.02, *SD* = 0.80; heterogeneous *M* = 2.73, *SD* = 0.84; high expertise *M* = 2.56, *SD* = 0.81), *F*(2,52) = 1.40, *p* = 0.257, $\eta_{p}^{2}$ = 0.054.

**FIGURE 2:**
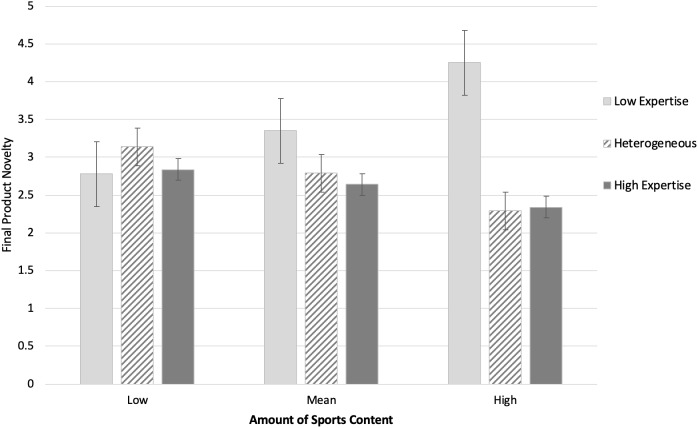
Final product novelty as a function of experimental condition (group composition) and amount of conventional sports content in the group’s final, convergent discussion phase.

Next, a moderation analysis was conducted to examine the effects of group composition on final product novelty as a function of conventional sports content in the convergent verbal interaction. For this analysis, we conducted a multi-categorical moderated regression equation using the Model 1 of the Process Macro for SPSS (Hayes, 2017) and 95% confidence intervals. Group composition was entered as the independent variable, final product novelty as the criterion variable, and sports content as the moderator. Indicator coding, with the high expertise group as the baseline, was used to test effects between groups, and the sports content moderator variable was mean centered. Contrast 1 compared the heterogeneous to the high expertise group; it was not significant. Contrast 2 compared the low expertise group to the high expertise group. The interaction between contrast 2 and the amount of sports content was significant, *b* = 1.44, *SE* = 0.71, *t*(6,42) = 2.05, *p* = 0.047, CI[0.02, 2.87]. At mean levels of sports content, lower final product novelty was found in high expertise group compared to the low expertise groups, *b* = 0.82, *SE* = 0.38, *t*(6,42) = 2.13, *p* = 0.039, CI[0.04, 1.59]. Likewise, at high levels of sports content, lower final product novelty was again found in the high expertise group compared to the low expertise groups, *b* = 1.97, *SE* = 0.85, *t*(6,42) = 2.32, *p* = 0.030, CI[0.25, 3.67]. As illustrated in Figure 2, these results suggest that the final product novelty in low expertise groups was enhanced via discussions using conventional sports words. For high and heterogeneous groups, by contrast, final product novelty tended to decrease as a function of conventional sports words usage. These results are supportive of our hypotheses concerning the roles of topical content, namely, that as expertise in a group increases, a fixation on expertise-relevant content can hinder novelty.

## Discussion

### Linking Divergent and Convergent Creativity

We believe this is the first study that has examined in detail the link between the various elements of the divergent and convergent phases of the collaborative creativity process. Our theoretical model (DCIM, Paulus et al., 2019) suggested that the key factors in linking the divergent and convergent phases would be the elaboration process of the convergent phase. Consistent with Hypotheses 1 and 2 we found that the number of replies to shared ideas and the novelty of those replies predicted the final product. The number of ideas and number of novel ideas did not predict this final outcome. These findings lend support to theoretical models that emphasize the importance of elaboration processes in collaborative creativity (van Knippenberg et al., 2004; Harvey, 2015; Paulus et al., 2019). To our knowledge, these are among the very few findings that link group processes during a divergent, ideation phase to a convergent product phase. These findings indicate that when group members engage with each other’s ideas, and build on them collaboratively, the final product can be enhanced.

Our findings indicated that the generation of novel ideas does not directly translate to a novel, final group product. This is consistent with research indicating there may be bias against novelty in the selection of ideas for implementation (Rietzschel et al., 2006; Putman and Paulus, 2009; Mueller et al., 2011). In some additional analyses of our data we found that the ideas that were rated as 3 or 4 on the 5- point novelty scale were somewhat more likely to obtain replies and votes relative to their actual frequency in the corpus. The most novel ideas (rated as 5) did gain the most votes and replies, but these were consistent with their frequency. The low rated ideas (rated as 1) were indeed recognized as reflected in the percentage of replies and votes relative to their frequency. Furthermore, when we traced the origins of the ideas incorporated into the highly rated final sports (rated 4 or 5), they were mostly ideas that were rated fairly low in novelty. Apparently, by combining these relatively low novelty ideas with other ideas, groups were able to develop novel sports. This is consistent with prior findings that groups may be particularly useful for the process of building on previously generated ideas (Kohn et al., 2011). We hope to explore these issues in more detail in future research.

Another potential reason that there was not a direct relationship between the novelty of the ideas and the final product novelty is the large number of ideas generated. If the divergent idea generation session were to be broken up into periodic evaluation sessions, it might be easier for groups to keep track of the most novel ideas (Harvey and Kou, 2013). Alternatively, it may be important to structure the convergent stage so that participants systematically list the ideas that received the most votes and replies prior to integrating the ideas into a final product.

Our study has thus demonstrated the importance of the elaboration process for the relationship between the divergent ideation phase and a subsequent convergent product development stage. As we argued above, reply novelty is likely an indicator of increased task engagement and the ideas that elicited novel replies are thus most likely to be salient to the group members and promoted during the product development phase. Thus even though there may be some degree of bias against novel ideas, our study has demonstrated that the novelty of the collaborative process does have a positive influence on the group innovation outcome.

### The Impact of Variation in Levels of Expertise

This study is also unique in its exploration of the role of levels of expertise. This factor did influence the divergent processes, but not the convergent outcome. In general, the groups with diverse levels of expertise generated the fewest ideas, and replies of the lowest average novelty, with the low expertise groups generating the most ideas and the most novel replies. These results provide partial support for Hypotheses 3 and 5b.

The fact that the heterogeneous groups performed most poorly is consistent with the idea that gaps in knowledge or interest can impede groups from taking advantage of their members’ diverse types or levels of knowledge in the innovation process. Although prior research has shown various benefits of diversity including an increase in the range of information, ideas, and suggestions exchanged (van Knippenberg and Schippers, 2007), our results illustrate some of the negative implications of diversity similar to the cognitive gaps found by Cronin and Weingart (2007). That is, the diversity of interest and experience in sports may have made it difficult to get into a semantic flow of ideas, which comes from building on each other’s ideas (Baruah and Paulus, 2011). Baruah and Paulus (2011) found that idea generation was enhanced when group members overlapped in their focus on a subset of categories rather than each focusing on a unique category. A natural follow-up to our present findings might be to systematically vary the degree of expertise heterogeneity within groups to examine at which point the divergence of expertise in a group becomes problematic for group productivity.

We also examined the endorsement of ideas within the divergent phase. We found that high expertise groups were less likely than other group types to vote for/endorse highly novel ideas during the divergent phase. They had a higher rate (compared to other group types) of endorsing non-novel ideas. This is consistent with the logic underlying hypothesis 4, namely, that high expertise groups would be constrained and inhibited by their pre-existing knowledge.

Supporting Hypothesis 4, the expertise level of the group predicted the amount of conventional sports related content discussed during the verbal interaction of the convergent phase. Consistent with the cognitive fixation reasoning (Wiley, 1998; Lewandowsky et al., 2007; Dane, 2010), the increased use of such words was associated with lower final product novelty for the high expertise groups but not for the low expertise groups. Thus for the high expertise and heterogeneous groups, but not the low expertise groups, the use of the conventional sports words inhibited their ability to incorporate novel elements into their final product. In our study the participants were not made aware of differences or similarity in expertise. We were interested in the natural processes of interaction among people who were similar or different in their respective levels of expertise. Of course, participants may have noticed the degree of similarity or difference in expertise over time, but research suggests that group members often have a difficult time determining the expertise of other group members (e.g., Littlepage et al., 1997; Baumann and Bonner, 2013). On group problem-solving tasks, increased recognition of expertise can enhance group performance and information sharing (Littlepage and Silbiger, 1992; Stasser et al., 2000; Baumann and Bonner, 2004). However, it may also be possible in the context of our study, which used an open-ended task, that recognition of expertise might have a negative effect on group interaction. The discovery that all group members are low in interest and experience in sports might have led to low expectations or goals. Learning of the diverse interest in expertise in heterogeneous groups may have led the low expertise members to defer to the high expertise members. Being aware that all group members have a high interest or expertise in groups might have had a positive motivational effect, but did not in the end translate to either greater productivity or novelty of ideas.

### Limitations and Future Directions

One typically mentioned limitation is the use of student populations in research. However, this type of highly controlled study involving intensive efforts over a period of 3 weeks would be nearly impossible to conduct with other populations such as those in work settings. Research on innovation in work settings relies mostly on survey measures because of time and cooperation constraints (Hülsheger et al., 2009; van Dijk et al., 2012). However, it might be useful to follow up this type of research with surveys on divergent and convergent processes in other settings, and possibly via case studies (e.g., Harvey and Kou, 2013). Moreover, the present finding concerning the importance of intellectual and social engagement in the collaborative innovation process has been evident in both laboratory and work settings (e.g., Argote and Levine, 2017; Guillaume et al., 2017), suggesting generalizability of our present findings.

Another limitation is the fact that our paradigm involved primarily asynchronous interactions instead of synchronous interactions. One reason for using the asynchronous approach is that it reflects the type of interaction that is typical in most innovation contexts, in that collective contributions are often spread out over time. It was also a more feasible approach for obtaining collaborative input over a period of 3 weeks. Although a number of other studies have used this approach and have found meaningful results (De Vreede et al., 2010; Coursey et al., 2018), it is possible that synchronous interaction may be more engaging. Thus a synchronous paradigm might lead to a stronger link between the content of the divergent phase and the convergent phase than an asynchronous one. An experimental design to test this proposal would employ both synchronous and asynchronous conditions, all else being held constant. Relatedly, our participants generated ideas and interacted via an online discussion board. Whereas we presumed that our participants would be familiar with various types of social interaction media, we did not measure such familiarity and thus could not assess the effects of potential differential levels of familiarity with online discussion boards.

Also, our final product task asked participants to focus on the various elements of the new sport they were proposing. It is possible that a task with fewer constraints might also show a clearer link between various features of the divergent phase, such as the novelty of ideas and number of replies. Thus future studies of the link between divergent and convergent creativity phases should use different paradigms and types of tasks to determine how these determine the nature and strength of the link. However, the fact that we found a link in our somewhat constrained paradigm suggests that other paradigms might find even stronger effects.

In our study, novel ideas were generally recognized (i.e., they received more votes) and novelty of elaborative replies influenced the final product. Thus our research suggests that, contrary to a potential bias against novel ideas, participants in our paradigm (especially those groups that were not composed of all high-expertise members) were able to overcome this bias. Future studies should examine the factors that determine the extent to which novel ideas obtain collaborative responses and gain momentum for inclusion in some final product. It could be that the asynchronous paradigm allowed for more reflection on the shared ideas, and thus enhanced the likelihood of building on those ideas. For example, Kohn et al. (2011) found when participants were presented ideas generated by others, groups but not individuals were able to use rare or novel ideas to generate even more novel ideas. In a similar vein, we recognize that we did not directly assess participants’ own views of the novelty of their own ideas. There is much past research using self-assessments, and we aimed to avoid the potential self-report biases inherent in such measurement by using independent trained coders to assess overall novelty of each idea, compared to the rest of the corpus of ideas obtained.

## Conclusion

This study has illustrated the importance of a number of dynamic group discussion elements in the innovation process. Specifically, the elaborations of others’ ideas and the novelty of such elaborations were key predictors of the novelty of the final product. Expertise levels also influenced aspects of the collaborative group discussions, with low expertise groups demonstrating the most ideational activity in the asynchronous interaction phase. In addition, the fixation of high expertise groups on sports words in the convergent phase inhibited their ability to develop novel sports. Thus, consistent with observations of creative interactions in real-world settings, group discussions appear to play an important role in creative outcomes. Our research has demonstrated the importance of specific aspects of the collaborative exchange process. In future studies, with the type of rich interactional data that we obtained in this discussion board type of research paradigm, it will be feasible to do even more detailed analyses of features of group discussions, such as synchrony (Dunbar and Mejia, 2013) and the extent to which group members persist in certain types of exchanges or patterns of discussions, such as intense exchanges within subgroups, or the depth of focus on specific topics or ideas.

## Ethics Statement

This study was approved by the Institutional Review Board of the University of Texas at Arlington. Participants were provided informed consent forms which clearly outlined the study procedures and their options.

## Author Contributions

LC, RG, BW, JK, PP, and SD involved in the design and conduct of the research. LC and RG took the lead in the major data analyses. SD provided supplementary analyses. The major writing was done by LC, RG, PP, and JK.

## Conflict of Interest Statement

The authors declare that the research was conducted in the absence of any commercial or financial relationships that could be construed as a potential conflict of interest.

## Footnotes

1 www.simplemachines.org

## APPENDIX A

### Script Read to Participants Prior to Discussing and Creating Final Product

“You will now communicate with your group members using Skype. Discuss the overall plan for your group sport and begin to make decisions. You should focus on finalizing your group’s sport. Remember, your sport should involve some physical activity, physical movement, or athletic component. Answer questions such as: How will your sport be structured? How many players? What objects/tools are used? What are the rules going to be? What are the time duration, physical space dimensions, and determinants of scoring? Etc. Your job is to work with the other members of your group to propose your new sport complete with an explanation of how the sport is played, the general structure, and rules of the game.

When you and your group members make a decision about your sport, record it on your word document. You will have 30 min to complete your sport and fill out your word document.”

### Template Document Given to Groups Prior to Discussing and Creating Final Product

#### New Sport Template

Sport Name:

Number of Players:

Objects used:

Location of Play:

Duration of Play (how long, minutes/hours/etc.):

Rules of Play (fouls, what is allowed, banned):

Point system used:

How do you earn points?:

How do you determine who wins?:

Extra Comments:

## APPENDIX B

### Heterogeneous Group, Final Product Novelty = 4.33

Participants created a novel sport known as “Live Action Chess” which consists of 2 teams of 13 for a total of 26 players. Players are on a large human sized chess board and donned with their respective armor that corresponds to their unique chess piece character (e.g., The knight wears an armor plate and holds a wooden lance, the King has a crown, cloak, and a hidden secret weapon). Players move in the same pattern as do chess pieces with the goal in mind to take the king. Points can also be accrued by taking over other enemy chess pieces. To ensure safety throughout the game, there will be four referees monitoring game play as well as beverages and snacks provided when players are not engaged in a move. Should a player intentionally harm someone or use an actual weapon that team is disqualified. Each team will have an allotted time out every hour as well as 2 emergency time outs. Each team only has 2 min to execute a move.

### Low Expertise Group, Final Product Novelty = 4.67

Participants created a novel sport entitled “Tennis Pong” which is comprised of 2 teams with 2 players. Players have 30 min to hit tennis balls with racquets over a net into large oversized cups (approximately 3 feet wide and 6 feet tall). Should a player hit another person with a tennis ball or fail to hit the ball over the next, that player automatically loses his or her turn. There are a total of 12 cups for each team, the team that eliminates the most cups of the opposing team wins the game. Should only one cup remain for a team both team members have to make it in to win.

### High Expertise Group, Final Product Novelty = 1.00

Participants formed a sport called “Katchball” which is modeled after dodgeball. Instead of using a dodgeball, the material would be similar to a kickball but be dodgeball sized. Play occurs on a tennis court without a net. This game can be played 1 vs. 1 or 2 vs. 2. Each game lasts 20 min and whichever team or player has one 3 out of 5 is determined the victor. Each side starts with one ball and points are awarded for catching the ball (3 points), hitting the opponent (2 points), and for dodging (1 point).

### Heterogeneous Group, Final Product Novelty = 1.00

Participants developed a new sport referred to as “Head Ball” which is essentially volleyball but players are only allowed to use their heads to hit the ball over the net. The sport takes place in an indoor volleyball court, where players are required to hit a volleyball with their head over a 7-foot net. Players play 4 ten-minute rounds with a 5-min half time in between. Points are accrued if the ball hits the ground (1 point), if any players use any other part of their aside from their heads (1 point), and if the ball goes outside the lines (3 points). Whichever team has the most points at the end wins the game.

#### Samples of Individual Ideas

• Jumping on the trampoline with a basketball in your hands and, as you are in mid air, releasing the ball to let it land in the basket.
• Consists of rollerblading toward a set of bowling pins while holding a bowling ball in your hands. then, the person releases the ball and lets the ball roll down the pathway to knock down the pins.
• Sticky hands and feet, contestants make their way through a 3D structure up to the top by adhering to its walls, could be magnetic or no.
• One can compete in how fast they build something out of scraps.
• Gear is similar to hockey and or football and all players must wear ice skates.
• How fast someone can run up some stairs with the stairs having some type of obstacles to go through. This race will be timed.
• Sodgeball: the combination of soccer and dodge-ball. Unlike soccer- the team will not consist of 11 but rather 6 people on a team similar to dodge ball. The rules are similar to dodgeball except that instead of throwing it, we can only kick the ball at other people. Rules are simple, if you get hit- you’re out. This continues until the opposing team has no more players. Each team starts off with only 3 balls. Unlike dodgeball- catching the opposing team’s ball does nothing except eliminate you, however, if you kick a ball that was kicked at you before it touches the ground and it hits a player on the opposing team then the player on the opposing team is eliminated – and if it hits the player who kicked it initially then that person is out and a person on your team who was out can come back in. Also if a ball happens to hit a player in the head or groin then the person who kicked the ball gets a red card and is out indefinitely for the remainder of the game. The game is best out of three rounds so two wins is necessary, players who are penalized for kicking the ball to the head or groin are still punished and cannot join the game for the second and third round.
